# Commentary: Chemokines in Prediabetes and Type 2 Diabetes: A Meta-Analysis

**DOI:** 10.3389/fimmu.2021.729702

**Published:** 2021-09-16

**Authors:** Jie Wei

**Affiliations:** Department of Hematology, People’s Hospital of Baise, Baise, China

**Keywords:** letter, chemokines, prediabetes, diabetes, meta

We read the paper by Pan et al. (1) with interest. The authors report a meta-analysis to summarize the results of previous studies on the association between the chemokines system and type 2 diabetes (T2DM)/prediabetes (PDM). They concluded that progression of T2DM may be associated with elevated concentrations of chemokines. After carefully reading, I wish to put forth the following suggestions.

References 12 (Sindhu et al., 2017) (2) and 102 (Sindhu et al., 2016) (3) in **Table 1** were created by the same author team, with the characteristics (Male gender, BMI, and Mean age) inserted wrongly. This wrong insertion of the data would affect the credibility of the meta-analysis’s final results. I recommend that the authors (1) must include both works (Sindhu’s papers) in the meta-analysis but a previous correction and reliable checking of the data insertion is required.

Not only in **Table 1** but also in **Figure 1**. In this figure, every insertion of the “Sindhu 2017” was wrong, instead “Sindhu 2017” should have been inserted as “Sindhu 2016”. Furthermore, in “type: CCL2”, the data for “Sindhu 2016” was inserted with 13 diabetic and 25 non-diabetic individuals when it must have been 53/72 or 23/24, depending on either Sindhu 2017 or 2016. Also, in “type: CCL11” and “type: CCL4” must be inserted both work’s data (2, 3).

Due to these mistakes, I recommend to the editor to request a completely new evaluation of all data setting, the correction of tables and figures, and between the data insertion and wrong data typing. Mistakes in the data typing are not allowed if the credibility of the meta-analysis results is desired.

## Author Contributions

The author confirms being the sole contributor of this work and has approved it for publication.

## Conflict of Interest

The author declares that the research was conducted in the absence of any commercial or financial relationships that could be construed as a potential conflict of interest.

## Publisher’s Note

All claims expressed in this article are solely those of the authors and do not necessarily represent those of their affiliated organizations, or those of the publisher, the editors and the reviewers. Any product that may be evaluated in this article, or claim that may be made by its manufacturer, is not guaranteed or endorsed by the publisher.
